# Supplementary material for: Type-specific dendritic integration in mouse retinal ganglion cells
Source: Nat Commun. 2020 Apr 30;11:2101. doi: 10.1038/s41467-020-15867-9 (PMC7193577; doi:10.1038/s41467-020-15867-9)
Supplement: Supplementary file 1 — Supplementary Information [file 41467_2020_15867_MOESM1_ESM.pdf]

SUPPLEMENTARY INFORMATION

Type-specific dendritic integration in mouse retinal ganglion cells

Ran, Huang et al.

- Supplementary Methods
- Supplementary Statistical Analysis
- Supplementary Figures
- Supplementary References

SUPPLEMENTARY METHODS

Receptive field (RF) estimation for simultaneous recorded electrical and Ca^{2+} imaging data

For binary spike data, RF (k) is characterized by a linear-nonlinear-Poisson (LNP) model,

$$\lambda(t) = f(\sum k_i x_{it}) \tag{1}$$

where k is the spatio-temporal RF (STRF), x is the binary dense noise stimulus, $y(t)$ is the spike count in a time bin of size Δ at time t , and f is a nonlinear function that ensures the spike rate is nonnegative, which is fixed to be a soft-rectification function. RF outputs were summed and passed through f to determine the conditional intensity $\lambda(t)$, which is then passed into a conditionally Poisson process to generate spike train:

$$P(y(t)|\lambda(t)) = \frac{1}{y(t)!}(\Delta\lambda(t))^{y(t)}e^{-\Delta\lambda(t)} \tag{2}$$

Since there is no ready-to-use evidence optimization method for LNP model, here we parameterized the STRF with a cubic regression spline basis[1, 2] to ensure smoothness. The number of basis functions used is chosen by cross-validation. Then, the model parameters (θ, weights on the basis functions for STRF) are fitted by maximizing the log-likelihood:

$$\mathcal{L}(\theta) = \sum_t \log\lambda(t) - \Delta\sum_t \lambda(t) \tag{3}$$

For both voltage and Ca^{2+} data, RF is characterized by a Linear-Gaussian model, as same as the one used for dendritic RFs in the main text. However, for a fair comparison with the spike RF, we fit STRF with the same spline basis by minimizing the mean-squared error cost function with L1 regularization:

$$\mathcal{L}(\theta) = \frac{1}{t}\sum(k^T x - y)^2 + \alpha\sum|\theta| \tag{4}$$

where y is either a voltage or a Ca^{2+} trace, and α is the regularization weight determined by cross-validation.

Correlation between firing rates and calcium signal increment

Firing rates is calculated by binning its binary spikes according to the light stimuli trigger time, with a bin size of Δ=200 ms. Ca^{2+} signals were also resampled to the trigger time, then the signal increment is calculated by passing the gradient of corresponding Ca^{2+} signal to a rectified linear unit function (ReLU).

The correlation between firing rates and Ca^{2+} signal increment is then quantified by the Pearson correlation coefficient (ρ).

SUPPLEMENTARY STATISTICAL ANALYSIS

Receptive field size vs. dendritic distance

To model the dependence of receptive field (RF) size as a function of dendritic distance, we used a Gaussian Generalized Additive Model (GAM) with factor cell type, a smooth term for each type as a function of dendritic distance and random factor cell id.

```
rf_size ~ type + s(soma_dist, by = type, k = 50) + s(exp_date, bs = "re")
```

We set the basis dimension k=50 to allow for sufficiently "wiggly" smooth terms. Inspection of the model fit indicated that this was high enough (using `gam.check`). The resulting model was fit using n=1,324 data points and yielded the following results:

```
Parametric coefficients:
              Estimate Std. Error t value Pr(>|t|)
(Intercept)    12.7394     0.9679  13.162  < 2e-16 ***
typesustained  -1.4720     2.2130  -0.665  0.50606
typemini alpha -2.2966     2.0233  -1.135  0.25656
typef-mini     -5.8777     2.0320  -2.893  0.00389 **
Approximate significance of smooth terms:
                               edf Ref.df      F  p-value
s(soma_dist):alpha transient 10.628 13.241 21.584  < 2e-16 ***
s(soma_dist):sustained        4.821  6.094  2.634 0.014754 *
s(soma_dist):mini alpha       1.599  2.015  7.568 0.000535 ***
s(soma_dist):f-mini           1.806  2.265  0.507 0.564331
```

Thus, the smooth terms for the tOff alpha RGC and the mini alpha RGC are highly significant, indicating non-random variation of receptive field size with dendritic distance.

Overall, the model explained 54.6% of the deviance.

Analysis of the pairwise differences between cell types indicated that the RF size of tOff alpha RGC dendrites close to the soma (< 50 to 75 µm) was significantly larger than that of other RGC types.

Receptive field offset vs. dendritic distance

To model the dependence of RF offset as a function of dendritic distance, we used a Gaussian GAM with factor cell type, a smooth term for each type as a function of dendritic distance and random factor cell id.

```
offset ~ type + s(soma_dist, by = type, k = 50) + s(exp_date, bs = "re")
```

We set the basis dimension k=50 to allow for sufficiently "wiggly" smooth terms. Inspection of the model fit indicated that this was high enough (using `gam.check`). The resulting model was fit using n=1,324 data points and yielded the following results:

```
Parametric coefficients:
              Estimate Std. Error t value Pr(>|t|)
(Intercept)     24.396      2.602   9.376  < 2e-16 ***
typesustained   23.990      6.039   3.973 7.51e-05 ***
```

```
typemini alpha   12.717      5.415   2.348    0.019 *
typef-mini        4.088      5.493   0.744    0.457
Approximate significance of smooth terms:
                                    edf Ref.df      F  p-value
s(soma_dist):typealpha transient  3.341  4.227 11.78 1.15e-09 ***
s(soma_dist):typesustained        9.686 12.062 53.49  < 2e-16 ***
s(soma_dist):typemini alpha       4.349  5.513 50.12  < 2e-16 ***
s(soma_dist):typef-mini           4.114  5.115 23.18  < 2e-16 ***
```

Thus, the smooth terms for all RGC types are highly significant, indicating non-random variation of RF centre offset with dendritic distance.

Overall, the model explained 63.2% of the deviance.

Receptive field overlap vs. dendritic distance and dendritic angle

To model the dependence of RF overlap as a function of dendritic distance and dendritic angle, we used a t-distributed GAM with 5 degrees of freedom. In this case, we found that allowing for t-distributed residuals improved the quality of the model (AIC: -61,825 vs. -59,202).

We included a factor cell type, a bivariate smooth term as a function distance and angle, unique for each type, and a random effect term for cell id.

```
overlap ~ type + te(roi_dist, angle, by=type, k=20) + s(cell_id, bs="re")
```

We set the basis dimension k=20. Inspection of the model fit indicated that this was high enough (using `gam.check`).

The resulting model was fit on n=54,194 data points and yielded the following results:

```
Parametric coefficients:
               Estimate Std. Error t value Pr(>|t|)
(Intercept)     0.53763    0.02234  24.063  < 2e-16
typesustained   0.28248    0.05067   5.575 2.49e-08
typemini alpha  0.20049    0.04854   4.130 3.63e-05
typef-mini      0.28648    0.04725   6.063 1.34e-09
Approximate significance of smooth terms:
                                     edf Ref.df       F p-value
te(roi_dist,angle):alpha transient 239.02 291.20 602.962  <2e-16 ***
te(roi_dist,angle):sustained        69.23  95.47   8.983  <2e-16 ***
te(roi_dist,angle):mini alpha      199.26 244.15  46.514  <2e-16 ***
te(roi_dist,angle):f-mini           14.02  19.21   1.884   0.011 *
```

This indicated that all cell types show significant variation in the smooth surface of RF overlap vs. angle and distance.

Overall, the model explained 72.8% of the deviance.

Correlations vs. dendritic distance and dendritic angle

To model the dependence of temporal correlation as a function of stimulus (local or global), dendritic distance and dendritic angle, we used a Gaussian GAM with factor cell type, stimulus, and a bivariate smooth term as a function distance and angle, unique for each combination of type and stimulus, and a random effect term for cell id.

```
corr ~ typeXchirp + te(roi_dist, angle, by=typeXchirp, k=50) + s(cell_id, bs="re")
```

We set the basis dimension k=50. Inspection of the model fit indicated that this was high enough (using `gam.check`).

The resulting model was fit on n= 33,796 data points and yielded the following results:

```
Parametric coefficients:
                                       Estimate Std. Error t value Pr(>|t|)
(Intercept)                            0.418720   0.053359   7.847 4.38e-15 ***
sustained.global                      -0.094504  0.105581  -0.895   0.3707
mini alpha.global                      0.213602   0.115928   1.843   0.0654 .
alpha transient.local                  0.033782   0.003665   9.217  < 2e-16 ***
sustained.local                        0.028753   0.105457   0.273   0.7851
mini alpha.local                       0.267182   0.115465   2.314   0.0207 *
Approximate significance of smooth terms:
                                          edf     Ref.df  F      p-value
te(roi_dist,angle):alpha transient.global 246.85  345.0  11.307  <2e-16 ***
te(roi_dist,angle):sustained.global       190.18  265.0  12.363  <2e-16 ***
te(roi_dist,angle):mini alpha.global      226.69  316.9  10.646  <2e-16 ***
te(roi_dist,angle):alpha transient.local  164.80  234.3  12.321  <2e-16 ***
te(roi_dist,angle):sustained.local        229.01  314.3   9.953  <2e-16 ***
te(roi_dist,angle):mini alpha.local       147.13  208.7   8.857  <2e-16 ***
s(cell_id)                                 14.96   16.0 928.261  <2e-16 ***
```

This indicated that all cell types show significant variation in the smooth surface of RF overlap vs. angle and distance for both local and global stimuli.

Overall, the model explained 62.8% of the deviance.

Correlation between Ca^{2+} signal increase and the number of spikes evoked by current injection

We used linear regression to analyse the relationship between Ca^{2+} signal increase and the number of spikes evoked by current injection across four groups: proximal and distal dendrites of tOff alpha and mini alpha.

```
dendriticCalcium_area ~ SomaticSpikes * group
```

The resulting model was fit on n=276 data points and exploratory ANOVA yielded the following results:

```
Analysis of Variance Table
Response: DendriticCalcium_area
                     Df Sum Sq Mean Sq F value    Pr(>F)
```

```
SomaticSpikes         1   6704  6703.7  24.716 1.190e-06 ***
group                 3  20207  6735.7  24.834 3.333e-14 ***
SomaticSpikes:group   3  22260  7420.1  27.357 1.851e-15 ***
Residuals           267  72418   271.2
Signif. codes:  0 '***' 0.001 '**' 0.01 '*' 0.05 '.' 0.1 ' ' 1
```

Thus the number of evoked spikes had a group specific impact on Ca^{2+} signal increase.

We computed the slope of the increase as a function of the number of evoked action potentials using `lstrends` for each group:

```
Group          SomaticSpikes.trend    SE  df  lower.CL upper.CL
toffa proximal               0.437 0.222 267 -0.00141    0.875
toffa distal                 0.238 0.110 267  0.02213    0.454
minia proximal               3.030 0.307 267  2.42595    3.635
minia distal                 1.402 0.283 267  0.84421    1.960
Confidence level used: 0.95
```

Then performed pairwise comparison between these slopes (using `pairs`)

```
contrast                        estimate    SE  df t.ratio p.value
toffa proximal - toffa distal      0.198 0.248 267  0.800  0.8542
toffa proximal - minia proximal   -2.594 0.379 267 -6.842  <.0001
toffa proximal - minia distal     -0.965 0.360 267 -2.680  0.0389
toffa distal - minia proximal     -2.792 0.326 267 -8.565  <.0001
toffa distal - minia distal       -1.164 0.304 267 -3.832  0.0009
minia proximal - minia distal      1.628 0.418 267  3.899  0.0007
P value adjustment: tukey method for comparing a family of 4 estimates
```

This shows that the slopes between all compared groups were significantly different ($p<0.05$) except the one between proximal and distal dendrites of tOff alpha.

SUPPLEMENTARY FIGURES

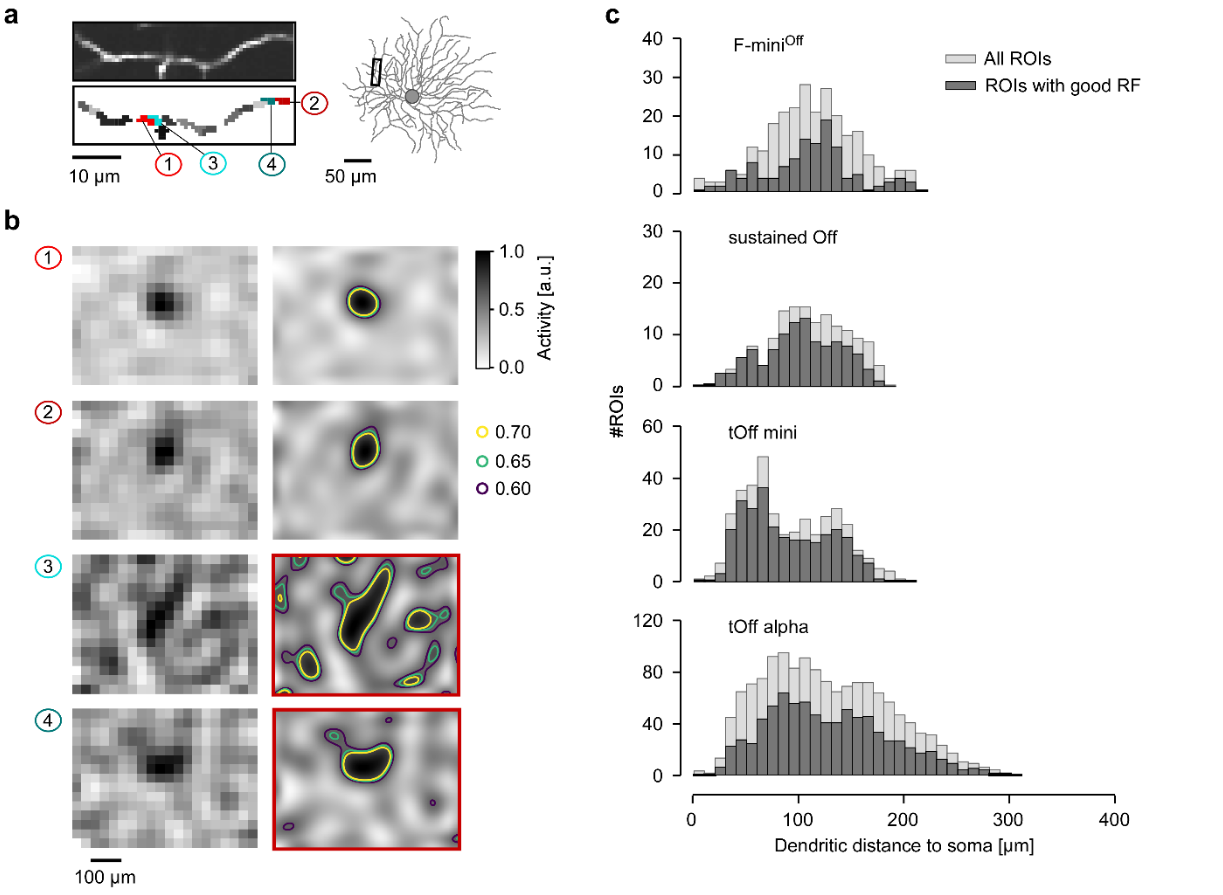

Supplementary Fig. 1 Region of interest (ROI) selection. **a**, Exemplary scan field (top left) and automatically generated ROI mask (bottom left) from the region labelled by black rectangle on the reconstructed RGC morphology (right). **b**, Smoothed and normalized RF maps before (left) and after (right) up-sampling for the labelled ROIs indicated in (a). Coloured curves on up-sampled RF maps (right) show RF contours with three different thresholds used for the RF quality test (Methods). Only ROIs that passed the test (a single contour with $\mathrm{Ii} < 0.1$, $A_{contour} > 1.8 \cdot 1{,}000$ µm^2, at a contour threshold of 0.60; see also Methods) were used for further analysis. Red rectangles around the up-sampled RF maps (right) indicate that RFs did not pass and were discarded. **c,** Histograms of recorded ROIs for tOff alpha (n=17\1,452\851 cells\total ROIs\ROIs that passed the RF quality test), tOff mini (n=5\387\295), sOff (n=4\208\154) and F-mini^{Off} RGCs (n=5\265\126).

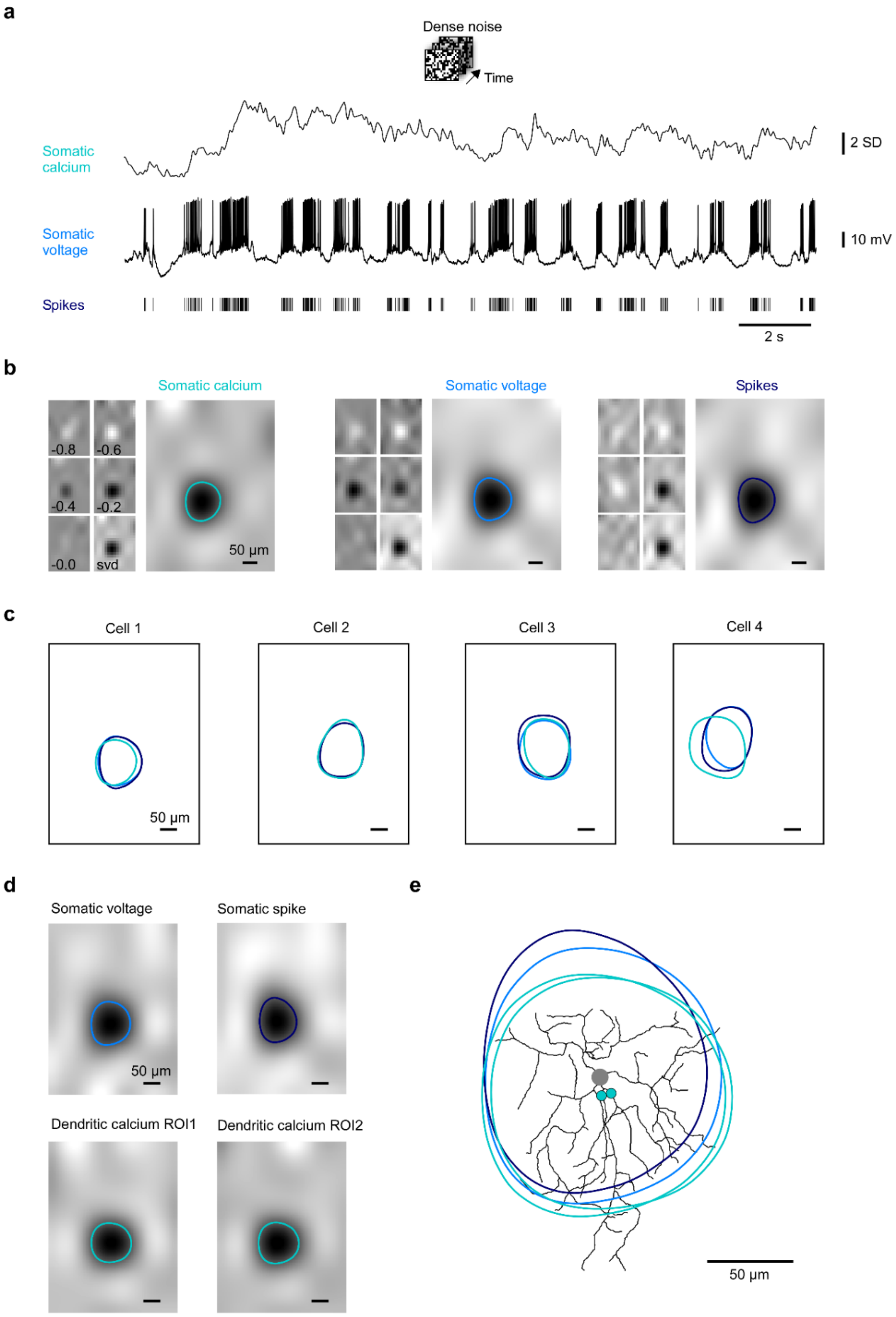

Supplementary Fig. 2 RFs estimated by simultaneous recordings of somatic voltage and somatic/proximal dendritic Ca^{2+} signals. For binary spike data, RF was characterized by a linear-nonlinear-Poisson (LNP) model; for both voltage and Ca^{2+} data, RF was characterized by a Linear-Gaussian model (see Supplementary Methods). **a,** Exemplary somatic voltage and Ca^{2+} traces in response to dense noise and the corresponding binary spike train normalized to the standard deviation (SD) of the baseline. **b,** RF maps estimated from somatic Ca^{2+}, voltage and spike rates at different times

(δ, [s]) before an event (small maps) and the up-sampled and smoothed singular value decomposition (SVD) maps with the RF contour. **c,** RF contours derived from somatic Ca^{2+}, voltage and spike rates in response to dense noise for n=4 cells. **d,** Up-sampled and smoothed RF maps with the corresponding RF contours for the soma and proximal dendrites from simultaneous somatic voltage recording and Ca^{2+} imaging from the proximal dendrites. **e,** RF contours from (d) and the proximal ROIs overlaid with the partially reconstructed cell morphology.

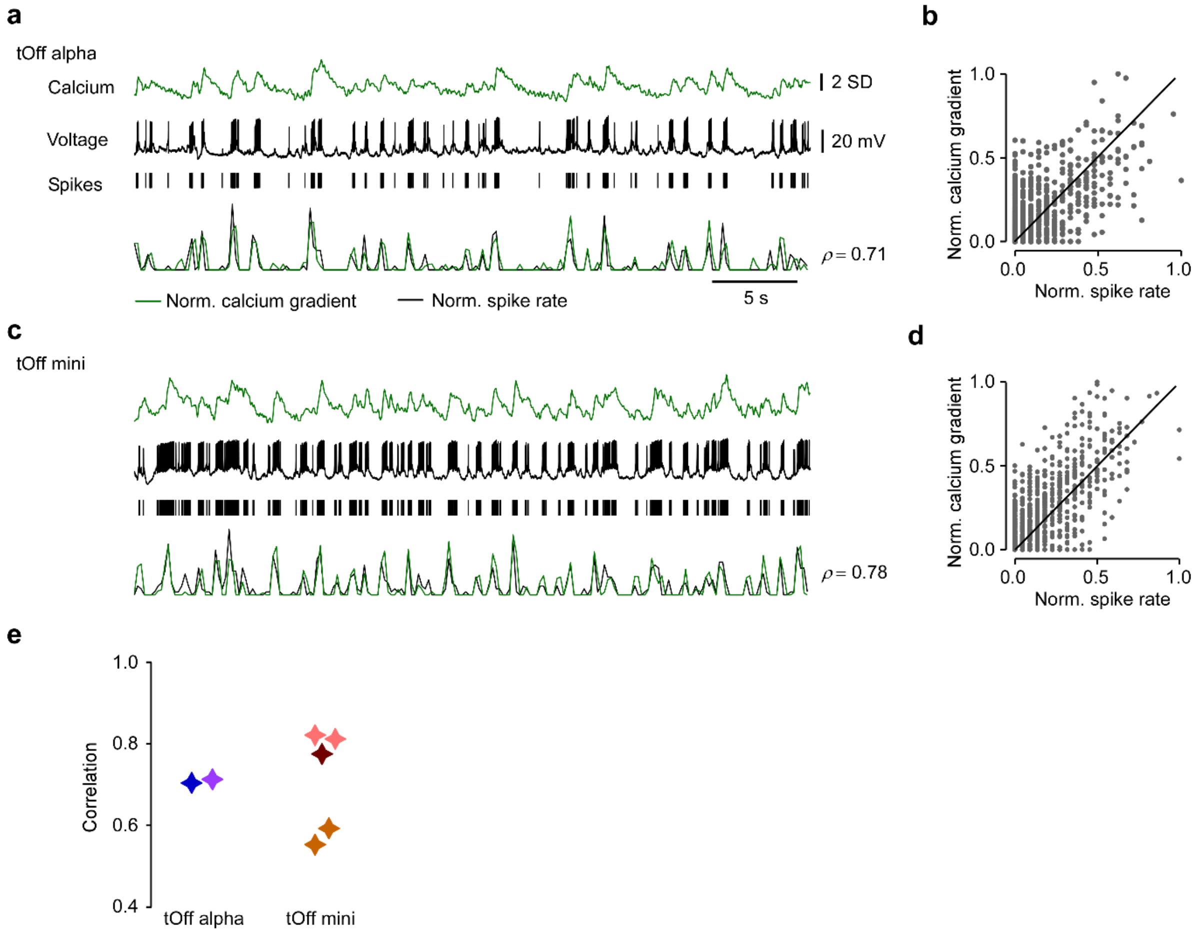

Supplementary Fig. 3 Relationship between spike rates and Ca^{2+} signal increments. **a,** Example for simultaneously recorded dendritic Ca^{2+} (proximal, 44 µm from soma) and somatic voltage signals from a tOff alpha cell to dense noise stimulation. Below the voltage trace, the extracted spike pattern and traces for normalized Ca^{2+} gradient (green) and normalized spike rates (black) are shown (values on the right from the overlaid traces indicate correlation between Ca^{2+} gradient and spike rates). **b,** Normalized Ca^{2+} gradient as a function of normalized spike rates. **c,** Like (a) but for a tOff mini cell (Ca^{2+} from proximal location, 26 µm from soma). **d,** Like (b) but for the tOff mini cell. **e,** Correlation between normalized Ca^{2+} gradient and normalized spike rates for n=2 tOff alpha and n=3 tOff mini cells (each star indicates one recording, colours indicate different cells; correlation range between 0.55 and 0.82).

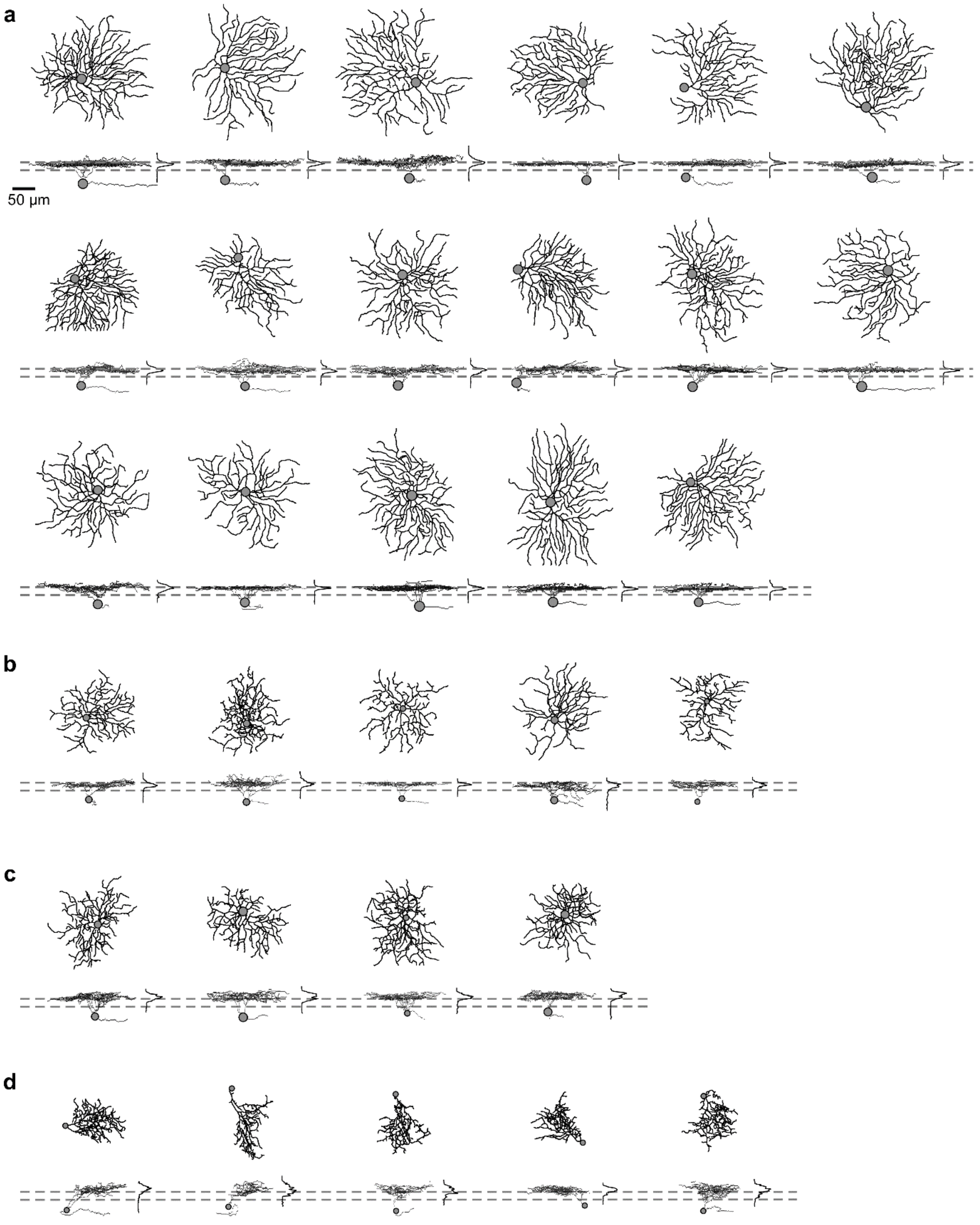

Supplementary Fig. 4 Morphologies of all recorded retinal ganglion cells (RGCs). **a**-**d**, Reconstructed morphologies clustered into RGC types using the algorithm published by Bae et al. (ref[3]): tOff alpha in (a), tOff mini in (b), sOff in (c) and F-mini^{Off} in (d).

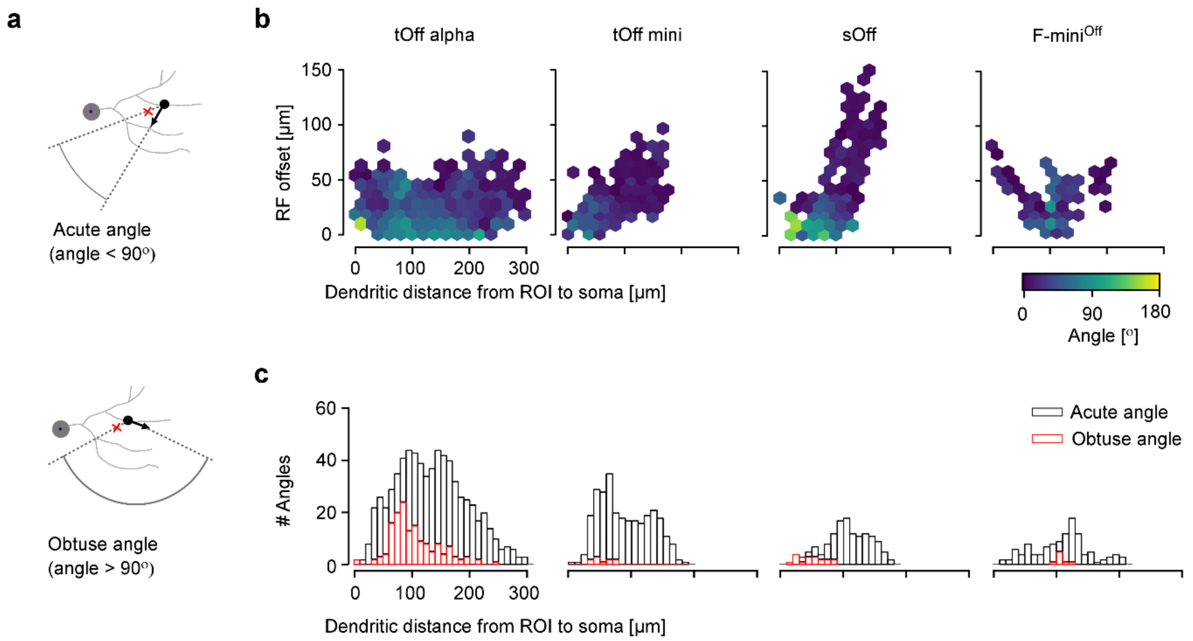

Supplementary Fig. 5 Quantification of RF offset angle. **a,** Illustration of the acute (top) and obtuse (below) angle measured between the lines from a ROI's centre to the dendritic arbour centre and a ROI's centre to its RF centre. Arrow points from the ROI´s centre to its RF contour centre. Red cross indicates the dendritic arbour centre. **b**, Color-coded RF offset angles as a function of dendritic distance to soma and distance from ROI to RF centre (RF offset). **c,** Distribution of acute (black) and obtuse (red) RF offset angles as function of dendritic distance from ROI to soma.

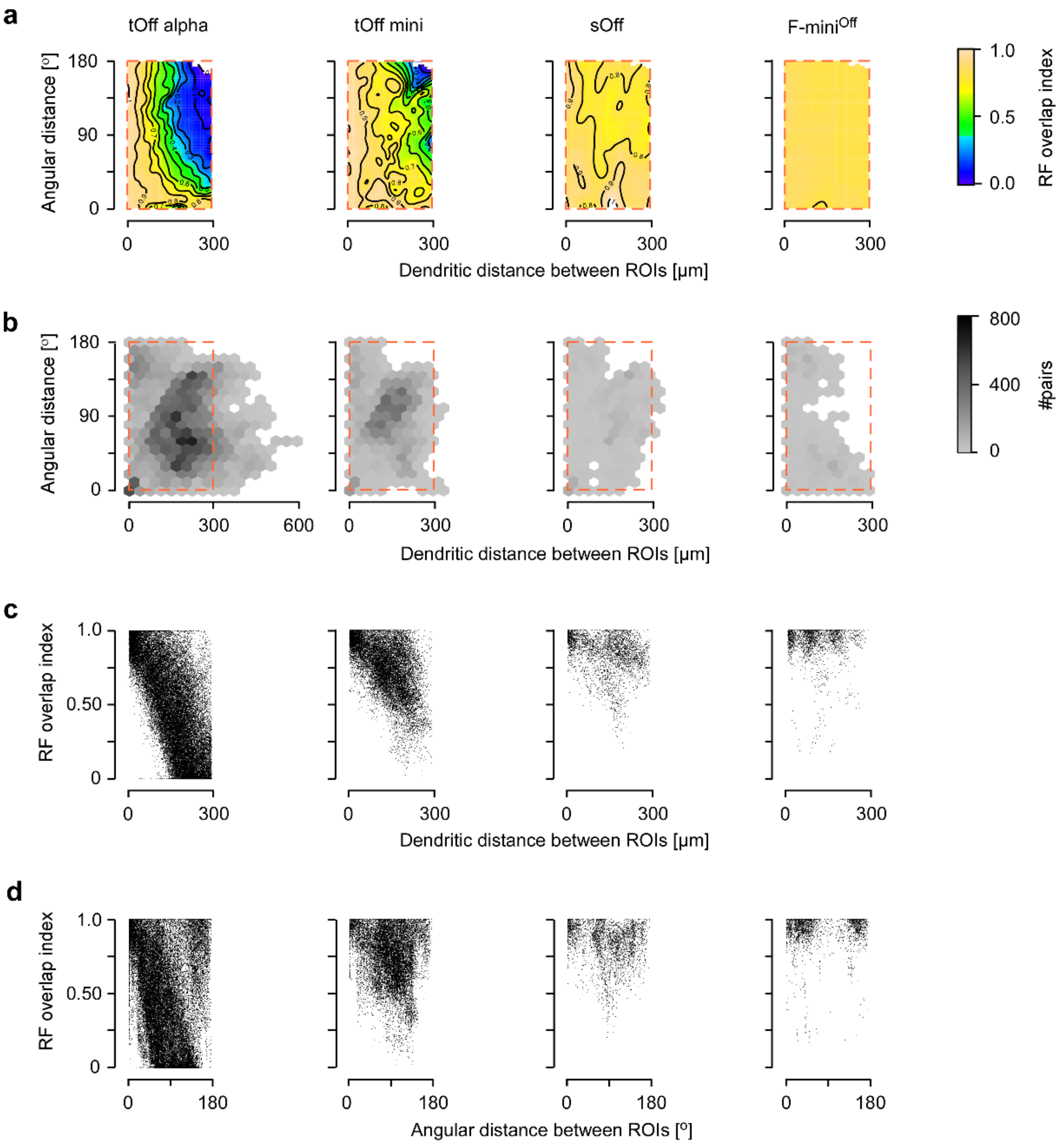

Supplementary Fig. 6 Dendritic RF overlap data used for statistical comparison. **a,** Generalized Additive Model (GAM) fitted maps (Methods) of the data shown in main Figure 4c for the plot area marked by dashed red rectangle; colours encode dendritic RF overlap index (Oi). **b,** Hexagon maps showing the number of ROI pairs available for estimation of the Oi in tOff alpha (n=17\40,777 cells\ROI pairs), tOff mini (n=5\13,524), sOff (n=4\3,141), and F-mini^{Off} RGCs (n=5\2,097). **c,** RF Oi as a function of dendritic distance between ROIs for the different RGC types, for same plot area as in (a). **d,** Like (c), but with RF Oi as a function of angular distance.

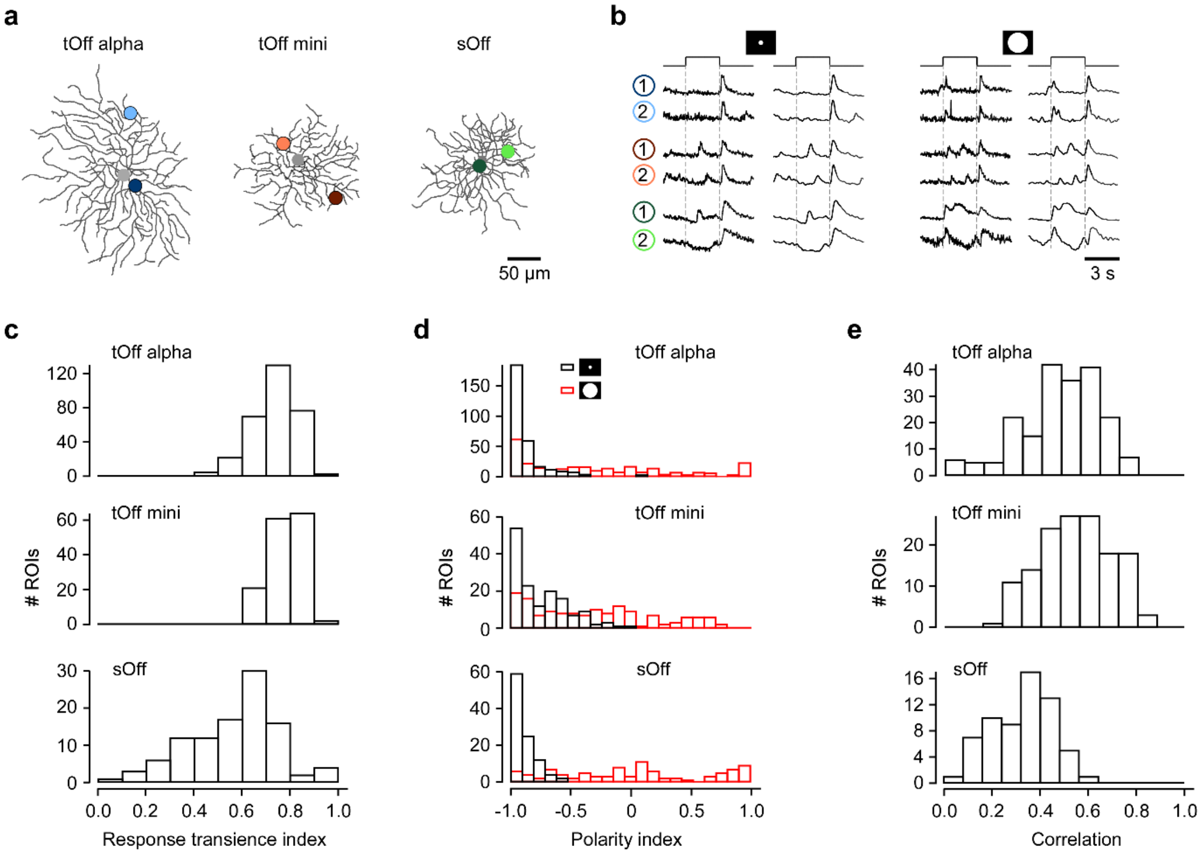

Supplementary Fig. 7 Temporal features of dendritic responses in different RGC types. **a**, Two ROIs overlaid with the reconstructed cells. **b**, Exemplary traces – unsmoothed and binomially smoothed (Methods) – in response to the step section of the local and full-field chirp stimulus recorded from the ROIs in (a). **c**, Histograms of response transience index (Ti, Methods) for tOff alpha (n=11\307 cells\ROIs), tOff mini (n=3\148), and sOff RGCs (n=4\103). **d,** Like (c), but with polarity index (POi) for tOff alpha (n=11\307\282 cells\ROIs for local\ROIs for full-field chirp), tOff mini (n=3\148\146), and sOff RGCs (n=4\103\93). **e,** Histograms of correlation between local and full-field chirp responses in tOff alpha (n=11\377 cells\ROIs), tOff mini RGCs (n=3\153), and sOff (n=4\129).

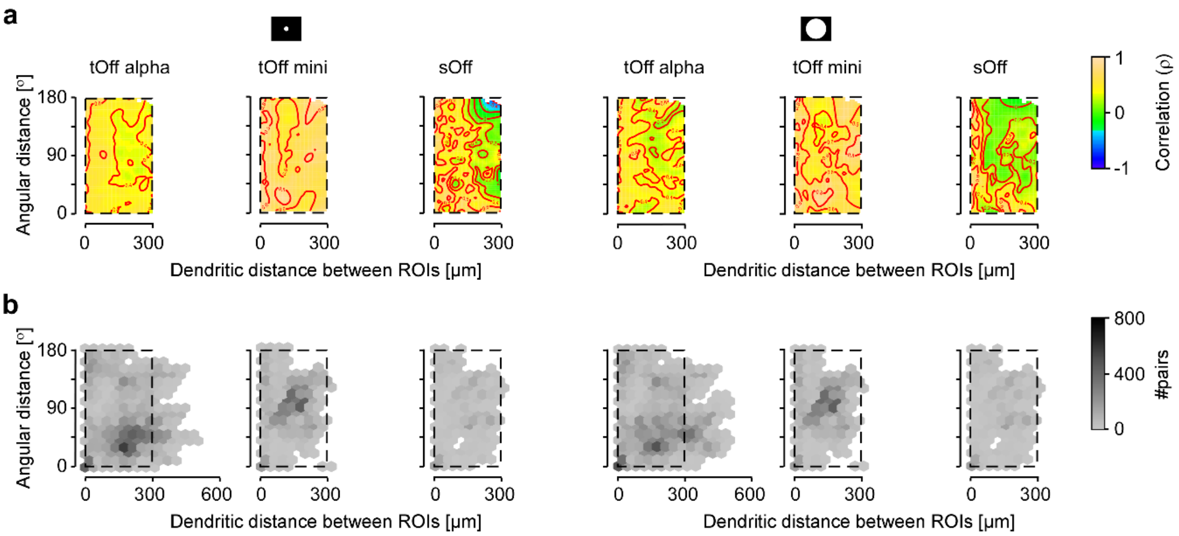

Supplementary Fig. 8 Local and full-field chirp correlation data used for statistical comparison. **a,** GAM-fitted maps (Methods) of the correlation data for local (left) and full-field chirp (right) responses shown in main Fig. 5d (for plot are marked by dashed black rectangle). **b,** Hexagon maps showing the number of ROI pairs available for estimation of correlation for local (left) and full-field chirp (right) responses.

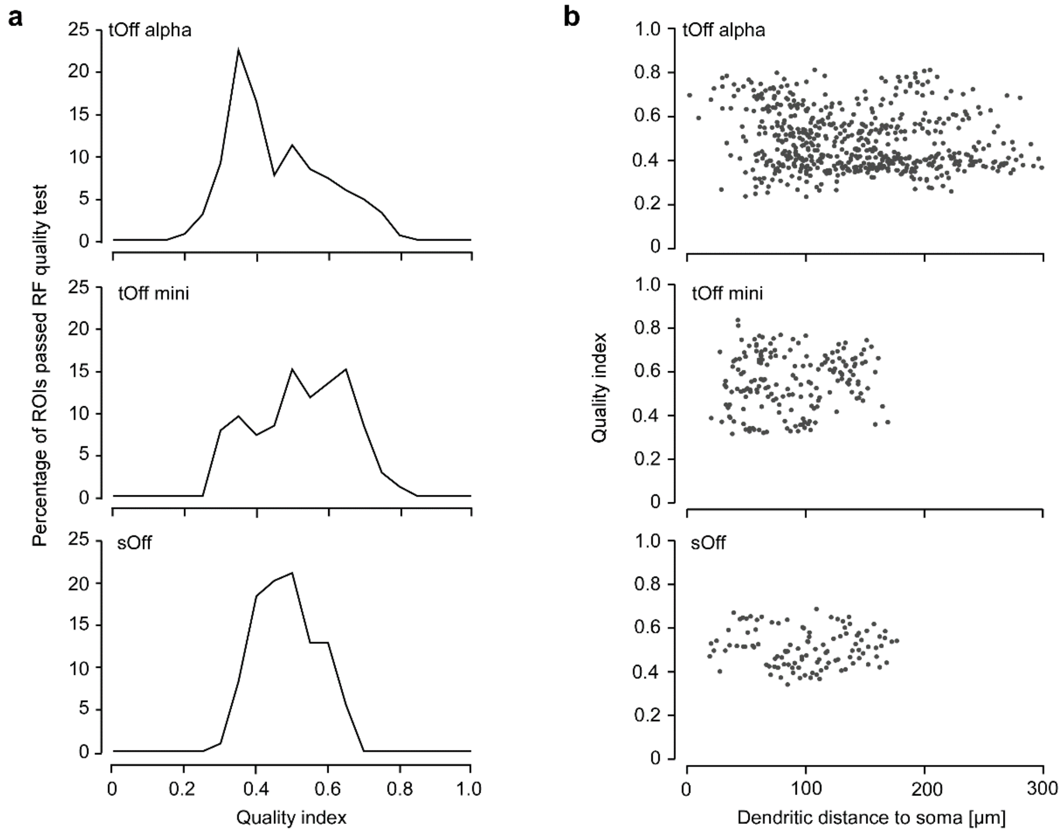

Supplementary Fig. 9 Quality index calculated from chirp responses in different RGC types. **a,** Histograms of Quality index. **b,** Quality index as a function of dendritic distance to soma.

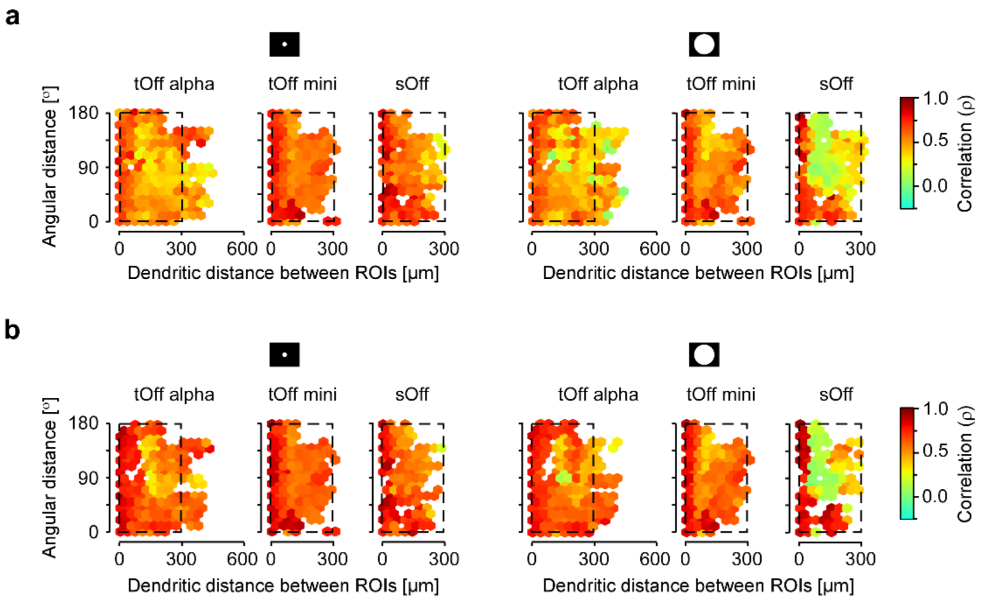

Supplementary Fig. 10 Temporal correlation across dendrites for different quality thresholds. **a,** Hexagon maps showing correlation calculated from dendritic signals evoked by local (left) and full-field chirp (right) as a function of angular distance and dendritic distance between ROIs for tOff alpha, tOff mini, and sOff RGCs. Colour encodes correlation; only ROIs with Qi > 0.4 included (Methods). **b,** Same as in (a), but for ROIs with Qi > 0.5. Dashed black rectangles indicate plot area used for statistical analysis (e.g. in main Fig. 5e-g).

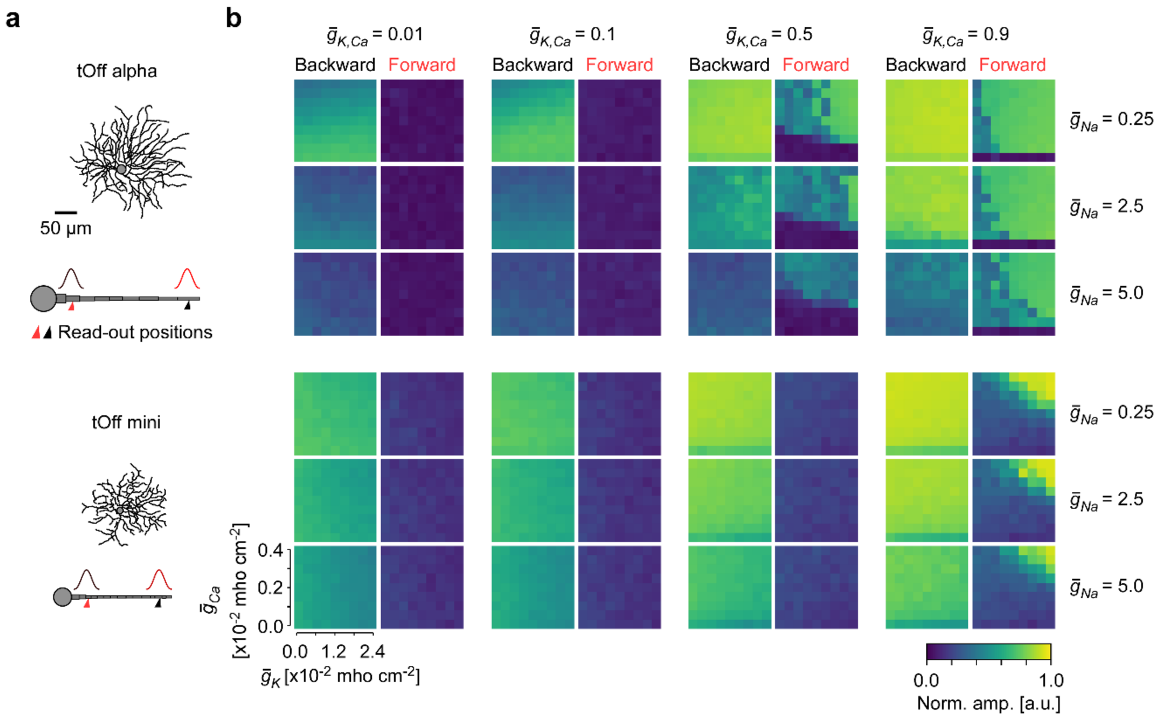

Supplementary Fig. 11 Simulation of dendritic signal propagation in tOff alpha and tOff mini RGCs. **a,** Reconstructed cell morphologies of a tOff alpha and a tOff mini RGC with illustrations of the respective ball-and-stick models (same as in main Figure 7a, c). Simulated inputs at proximal (25 µm to soma) and distal (85% of the total dendrite length to soma) positions indicated as red and black Gaussians, respectively. **b,** Heat maps showing the signal amplitude at the two read-out positions indicated in (a), normalized to the amplitude at the respective input position as a function of ion channel density combinations.

SUPPLEMENTARY REFERENCES

1. Wood SN. *Generalized additive models: an introduction with R*. (Chapman & Hall, Boca Raton, Florida, U. S. A., 2006).

2. Huang Z, Berens P. Efficient high dimensional receptive field inference using a flexible spline basis. Cosyne Abstracts 2020, Denver, CO (2020).

3. Bae JA*, et al.* Digital Museum of Retinal Ganglion Cells with Dense Anatomy and Physiology. *Cell* 173, 1293-1306 e1219 (2018).
