# Supplementary material for: Type-specific dendritic integration in mouse retinal ganglion cells
Source: Nat Commun. 2020 Apr 30;11:2101. doi: 10.1038/s41467-020-15867-9 (PMC7193577; doi:10.1038/s41467-020-15867-9)
Supplement: Supplementary file 3 — Reporting Summary [file 41467_2020_15867_MOESM3_ESM.pdf]

nature research

Corresponding author(s): Thomas Euler

Last updated by author(s): Mar 16, 2020

Reporting Summary

Nature Research wishes to improve the reproducibility of the work that we publish. This form provides structure for consistency and transparency in reporting. For further information on Nature Research policies, see Authors & Referees and the Editorial Policy Checklist .

Statistics

For all statistical analyses, confirm that the following items are present in the figure legend, table legend, main text, or Methods section.

n/a	Confirmed	
☐	☒	The exact sample size (*n*) for each experimental group/condition, given as a discrete number and unit of measurement
☐	☒	A statement on whether measurements were taken from distinct samples or whether the same sample was measured repeatedly
☐	☒	The statistical test(s) used AND whether they are one- or two-sided<br>*Only common tests should be described solely by name; describe more complex techniques in the Methods section.*
☐	☒	A description of all covariates tested
☐	☒	A description of any assumptions or corrections, such as tests of normality and adjustment for multiple comparisons
☐	☒	A full description of the statistical parameters including central tendency (e.g. means) or other basic estimates (e.g. regression coefficient) AND variation (e.g. standard deviation) or associated estimates of uncertainty (e.g. confidence intervals)
☐	☒	For null hypothesis testing, the test statistic (e.g. *F*, *t*, *r*) with confidence intervals, effect sizes, degrees of freedom and *P* value noted<br>*Give P values as exact values whenever suitable.*
☒	☐	For Bayesian analysis, information on the choice of priors and Markov chain Monte Carlo settings
☐	☒	For hierarchical and complex designs, identification of the appropriate level for tests and full reporting of outcomes
☐	☒	Estimates of effect sizes (e.g. Cohen's *d*, Pearson's *r*), indicating how they were calculated

Our web collection on statistics for biologists contains articles on many of the points above.

Software and code

Policy information about availability of computer code

Data collection	ScanM (v2.04), IGOR Pro (v6), pClamp (v10.6), QDSpy (v0.77)
Data analysis	Python (v3.6.7), R (v2.3), Scikit-learn (v0.20.0), Scikit-Image (v0.14.2), itsadug (v2.3), mgcv (m1.8-24), emmeans (v1.4.4), NEURON (v7.7.0); and other custom scripts are available at https://github.com/berenslab/rgc_dendrites

For manuscripts utilizing custom algorithms or software that are central to the research but not yet described in published literature, software must be made available to editors/reviewers. We strongly encourage code deposition in a community repository (e.g. GitHub). See the Nature Research guidelines for submitting code & software for further information.

Data

Policy information about availability of data

All manuscripts must include a data availability statement. This statement should provide the following information, where applicable:
- Accession codes, unique identifiers, or web links for publicly available datasets
- A list of figures that have associated raw data
- A description of any restrictions on data availability

All relevant data are available at https://doi.org/10.5281/zenodo.3708064 .

Field-specific reporting

Please select the one below that is the best fit for your research. If you are not sure, read the appropriate sections before making your selection.

☒ Life sciences ☐ Behavioural & social sciences ☐ Ecological, evolutionary & environmental sciences

Life sciences study design

All studies must disclose on these points even when the disclosure is negative.

Sample size	We did not perform prior sample size calculation. As the experiments are very hard to do, we simply used all cells we could obtain in a reasonable time frame. We made sure, each cell type was sufficiently covered with >3 cells per type.
Data exclusions	We did not include RFs of noisy ROIs into the analysis as described in the Methods. In addition, we sometimes recorded data from other cell types (e.g. ON RGCs) which are not described here.
Replication	We consider recordings from each cell as an independent experiment, and all example figures reflect $n \geq 3$ biologically independent replicates.
Randomization	There are no experimenter defined experimental groups in this study, so no randomization was performed. The recorded cells were classified as one of four types based on morphological criteria. The type definition was derived from a previous study by Bae et al.
Blinding	The investigators were not blind to the cell type during recording, but only knew that the cell was an Off retinal ganglion cell of particular size. The specific cell type was determined by post hoc clustering using only cell morphological features by an analyst not doing the experiments.

Reporting for specific materials, systems and methods

We require information from authors about some types of materials, experimental systems and methods used in many studies. Here, indicate whether each material, system or method listed is relevant to your study. If you are not sure if a list item applies to your research, read the appropriate section before selecting a response.

Materials & experimental systems

n/a	Involved in the study	
☒	☐	Antibodies
☒	☐	Eukaryotic cell lines
☒	☐	Palaeontology
☐	☒	Animals and other organisms
☒	☐	Human research participants
☒	☐	Clinical data

Methods

n/a	Involved in the study	
☒	☐	ChIP-seq
☒	☐	Flow cytometry
☒	☐	MRI-based neuroimaging

Animals and other organisms

Policy information about studies involving animals; ARRIVE guidelines recommended for reporting animal research

Laboratory animals	Mice used in this study were purchased from Jackson Laboratory and housed under 22 degree Celsius , 55% humidity and a standard 12 hour day/night cycle. For all experiments, mice aged 5-8 weeks of either sex were used. We used the transgenic mouse line B6;129P2-Pvalbtm1(cre)Arbr/J ("PV", JAX 008069, The Jackson Laboratory, Bar Habor, ME) cross-bred with the red florescence Cre-dependent reporter line Gt(ROSA)26Sortm9(CAG-tdTomato)Hze ("Ai9tdTomato", JAX 007905). For some recordings, we also used the wild-type line (C57Bl/6J, JAX 000664).
Wild animals	The study did not involve wild animals.
Field-collected samples	The study did not involve field-collected samples.
Ethics oversight	All animal procedures were approved by the governmental review board (Regierungspräsidium Tübingen, Baden-Württemberg, Konrad-Adenauer-Str. 20, 72072 Tübingen, Germany) and performed according to the laws governing animal experimentation issued by the German Government.

Note that full information on the approval of the study protocol must also be provided in the manuscript.
